# Formation and Inhibition of Lipid Alkyl Radicals in Roasted Meat

**DOI:** 10.3390/foods9050572

**Published:** 2020-05-04

**Authors:** Yingjie Bao, Yuxia Zhu, Xiaopu Ren, Yawei Zhang, Zengqi Peng, Guanghong Zhou

**Affiliations:** 1 College of Food Science and Technology, National Center of Meat Quality and Safety Control, Nanjing Agricultural University, Nanjing 210095, China; byjdkw@163.com (Y.B.); 2016208019@njau.edu.cn (Y.Z.); 2016208010@njau.edu.cn (X.R.); zhangyawei@njau.edu.cn (Y.Z.); ghzhou@njau.edu.cn (G.Z.); 2 Synergetic Innovation Center of Food Safety and Nutrition, Nanjing 210095, China

**Keywords:** antioxidants, electron spin resonance, lipid oxidation

## Abstract

Free radicals are ubiquitous in roasted foods. In this work, lipid-derived carbon-centered alkyl radical formation was first studied in roasted meat by electron spin resonance (ESR). The influence of antioxidants on the inhibition of free radicals was investigated. The results showed that the high temperature, high heat transfer rate, and high polyunsaturated fatty acid (PUFA) content resulted in high radical content in roasted meat, while the high water content in meat retarded radical formation. The 0.03% addition of tea polyphenols (TPP) significantly reduced radical formation during roasting (*p* < 0.05), whereas the 0.03% rosemary extract (RE) had no significant inhibitory effect (*p* > 0.05). These results suggested that water retention and the addition of TPP would decrease radical generation during the roasting of meat.

## 1. Introduction

Free radicals have received a great deal of attention due to the close connection of their reactions with human ageing and various diseases [1,2]. In food, free radicals are derived from major food components or their reactive constituents, such as proteins, lipids, and carbohydrates. The formation and decay of free radicals lead to chemical changes in food and thus affect food quality during processing and storage [3].

Roasting is a popular cooking method that uses high temperatures (≥150 °C). Roasted foods properly cooked with optimum time and temperature have complex flavors and aromas. However, free radicals are ubiquitous in roasted foods. Many researchers have reported the radical formation in roasted foods, such as linseeds and peanuts [4], coffee beans [5], and wheat seeds [6]. However, few studies have attempted to evaluate free radical formation in roasted meats. In fact, the lipids in meat are prone to oxidation to generate carbon-centered lipid alkyl radicals (L∙), oxygen-centered lipid peroxyl radicals (LOO∙), and alkoxyl radicals (LO∙) in the initiation and propagation stages [7]. However, most studies concentrated on nonradical products in the termination stage such as thiobarbituric acid reactive substance (TBARS) content to evaluate lipid oxidation. Radical formation, as an early event in the lipid oxidation process, was rarely considered in roasted meats. In addition, lipid radical formation is considered to be associated with common harmful substance formation, such as polycyclic aromatic hydrocarbons [8] and heterocyclic aromatic amines [9]. Therefore, it is necessary to study the variation in lipid radicals in meat during roasting.

Tea and rosemary extracts are known as efficient natural antioxidants to prevent lipid oxidation in the food industry [10,11]. The antioxidant activities of tea and rosemary are associated with the presence of phenolic compounds, which can break the lipid radical chain reaction and thus inhibit lipid oxidation [12]. However, most studies have focused on scavenging free radical activity *in vitro* models, such as 2,2-diphenyl-1-picrylhydrazyl (DPPH) free radical scavenging capacity [13,14], and secondary oxidation products such as TBARS [15,16] to evaluate the antioxidant capacity. Few studies have directly evaluated the scavenging lipid radical capacity of tea and rosemary *in situ* in roasted meat.

Electron spin resonance (ESR), also called electron paramagnetic resonance (EPR), is a technique to directly and specifically detect chemical species with unpaired electron(s) such as free radicals [17]. ESR has been widely applied to detect radical formation in food research [18,19,20]. The objective of the present work is to investigate the influences of roasting conditions and water content on the free radical formation in roasted beef by using ESR. The effect of TPP and rosemary extract additives on free radical inhibition was also studied. *N-tert*-butyl-α-phenylnitrone (PBN) was used as a spin trap to characterize the detected radical species in roasted meat. The findings of this study provide a basis for reducing radical formation in roasted meat.

## 2. Materials and Methods

### 2.1. Chemicals

Spin-trap PBN (purity above 99.5%) for ESR spectroscopy and 2,2,6,6-tetramethyl-1-piperidinyloxy (TEMPO, 99%) were purchased from Sigma-Aldrich (Steinheim, Germany). PBN was dissolved in anhydrous alcohol, while TEMPO was dissolved in doubly distilled water. TPP (catechin > 98%, Yuanye, Shanghai, China) and a commercial lipid soluble rosemary extract containing 60% carnosic acid (Zelang, Nanjing, China) were used as natural antioxidants. 2-[*N*-morpholino]ethanesulfonic acid (MES) was supplied by Solarbio (Beijing, China) and dissolved in doubly distilled water. Then, a 1% NaOH water solution was used to adjust the pH value to 5.7.

### 2.2. Preparation of Meat Sample

*Preparation of sliced beef.* Beef was used to evaluate the effects of heating conditions on the radical formation. Fresh beef (rib eye) was cut into 3 × 5 × 0.25 cm pieces. Fifty-seven pieces of sliced beef were randomly divided into 19 groups and each group had 3 samples. One raw group was used as a control, and the other groups were heated under the following conditions: (1) effects of the heating method and time: four groups were grilled in tinfoil using a griddle (JD30A846, Supor, China), four were barbecued using an electric tabletop grill (HX-280, Vnash, China), and four were roasted in an electric oven (D3-256A, Toshiba, Japan) under the same conditions of 200 °C for 10, 15, 20 and 25 min; (2) effects of the heating temperature and the presence of water: three freeze-dried groups and three raw groups were roasted at 120, 160 and 200 °C for 20 min. For the freeze-drying treatment, the sliced beef samples were freeze-dried at −63 °C (LGJ-10C, Four-ring Science, China). The water contents of freeze-dried and raw beef were detected as 1.53% and 78.63%, respectively [21].

*Preparation of meat patties.* Raw pork, chicken breast, chicken thigh and beef were used to compare the differences in of radical formation among meat species during roasting. Each meat was minced by a meat mincer (TS8, FAMA, Italy) and randomly divided into three groups: 30 g meat slurries with 0.03% TPP, with 0.03% RE, and without antioxidant as a control. Each group had 3 samples. After stirring, the slurries were molded using a culture dish (6 × 1.5 cm; diameter × thickness) and then roasted by meat type at 200 °C for 20 min.

For the roasting treatment, the meat samples were placed in a preheated oven at the designated temperature. For the grilling and barbecuing treatments, the meat samples were flipped once at half of the designated time, and the temperature of the griddle surface or grill was measured with an infrared thermometer (Raytek, MT 4, Santa Cruz, CA, USA). After heating, the meat samples were cooled to room temperature for further experiments.

### 2.3. Preparation for Radical Determination by ESR

For ESR direct measurement samples (solid), the sliced beef roasted at 160 °C for 20 min was lyophilized at −63 °C for 36 h, and then ground to powder for ESR measurement [18]. The lyophilized raw beef powder was used as a control.

For ESR spin-trapping measurement (liquid), based on the previous description [22] with small modifications, a 3.0 g ground roasted beef was suspended in 28.5 mL of 50-mM MES buffer (pH 5.7) and 1.5 mL of 0.4-M PBN, and the solution was homogenized at 8000 rpm for 1 min (T25, IKA, Germany). The homogenates were immediately incubated at 55 °C for 1 h in a water bath and then rapidly cooled in an ice bath. Then, the homogenate suspension was filtered to obtain the filtrate for ESR measurement. The raw beef filtrate was prepared with the same process as a control, and the buffer with PBN was also detected as a blank.

### 2.4. Radical Standard Curve of TEMPO

Based on the description of Bolumar et al. [23], solutions of different concentrations of TEMPO were prepared (0.5, 1, 2, 5, 10 and 20 μM) with 50-mM MES buffer (pH 5.7) for the ESR measurement to obtain a calibration curve of free radical content. ESR analysis of each sample was carried out in duplicate. The ESR spectra of TEMPO showed an equidistant three-line signal with approximate intensities of 1:1:1. The concentration (x) was used as the abscissa, and the double integral area of the recorded first derivative ESR signal of the second peak (y) was used as the ordinate to calculate the linear regression equation. The linear regression equation was y = 0.66x − 0.28 (*n* = 6, R = 0.998). The mean of the center field doublet was double-integrated to calculate the radical concentration [24].

### 2.5. ESR Measurement

For the solid samples, approximately 0.6 g of analytical powder was put into a plastic pipe (diameter 0.5 cm) that was then gently tapped against the table to obtain a uniform system. For the liquid samples, 60 μL of analytical solution was absorbed into a glass capillary tube (diameter: 0.1 cm), and the end of the capillary was sealed with plasticine. The plastic pipes and glass capillaries were transferred to a cylindrical quartz tube and subsequently placed in the ESR cavity for measurement by an A 300–10 ESR spectrophotometer (Bruker, Rheinstetten, Germany) at room temperature. Each sample was analyzed in duplicate. The ESR settings were as follows: microwave power of 20 mW, sweep width of 100 G, modulation amplitude of 2.0 G and modulation frequency of 100 kHz. The g-factor was calculated using the expression:

*g* = *hv*/Hβ (1)

where *h* is Planck’s constant, *v* is the frequency, H is the magnetic field (G) and β is the Bohr magneton. Based on the theory of spin trapping, nitroxide nitrogen yields 1:1:1 triplet splitting, which is used to calculate a_N_. The β-hydrogen splits each of the nitrogen couplings further into a 1:1 doublet, which is used to calculate a_H_ [17].

### 2.6. Total Antioxidant Capacity (T-AOC)

T-AOC was measured using commercial kits (Nanjing Jiancheng Bioengineering Institute, Nanjing, China). The principle is that ferric ions in the reaction mixture can be reduced by antioxidant reducing agents, and then a blue complex of Fe^2+^-TPTZ(2,4,6-tri(2-pyridyl)-s-triazine) is generated. The absorbance was determined at 520 nm. One unit (U) of T-AOC is defined as the amount that increased the absorbance by 0.01 at 37 °C. Each sample was analyzed in triplicate. T-AOC values were expressed as unit/mg protein.

### 2.7. Statistical Analysis

A two-way analysis of variance (ANOVA) with interaction was performed to determine the significance of radical content, and Duncan’s multiple range tests were applied to ascertain differences among means using SPSS Statistics 19 (Chicago, IL, USA). The results are presented as the mean ± standard deviation. Differences with *p* < 0.05 were considered statistically significant. The figures were designed using Origin 8.0 (MicroCal, Northampton, MA, USA).

## 3. Results and Discussion

### 3.1. Characterization of Formed Radicals in Roasted Beef

**Figure 1:**
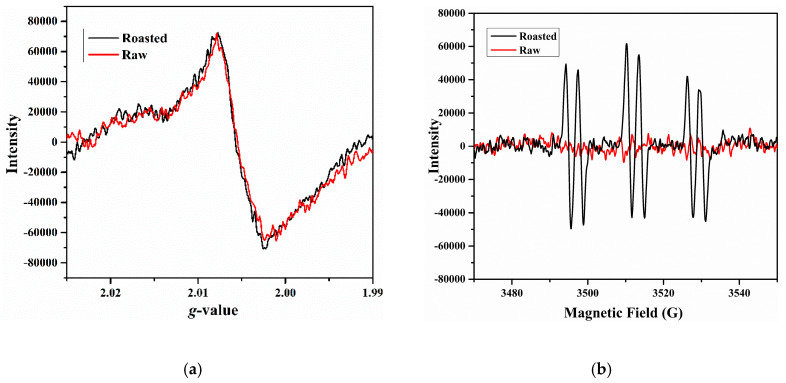
Electron spin resonance (ESR) spectra of roasted meat at 160 °C for 20 min. (**a**) Solid powder from lyophilized roasted beef and lyophilized raw beef; (**b**) filtrate of roasted and raw beef incubated at 55 °C for 1 h with PBN.

To characterize the formed radicals in roasted beef, the beef samples roasted at 160 °C for 20 min were treated with two independent preparations, including lyophilization for solid analytical samples and PBN spin trap for liquid analytical samples; the ESR spectra are shown in Figure 1. In general, the radical signal shape and intensity were obviously different for the solid and liquid groups, indicating that ESR measurement is sensitive to analytical sample states.

The solid analytical sample from lyophilized roasted beef powder produced a single broad line with a *g*-value of 2.0056, as shown in Figure 1a. This single-peak spectrum was similar to previous studies of solid samples [8]. Lyophilization has the advantage of removing tissue water and thus improves ESR measurement sensitivity. However, it was reported that lyophilization could produce free radicals in samples, resulting in a “lyophilization signal” in the ESR spectrum, which may arise from ascorbic acid [25]. A lyophilized raw beef powder was therefore prepared to verify the cause of the detected radical. As shown in Figure 1a, the lyophilized raw beef gave a similar ESR spectrum to the lyophilized roasted beef due to an equivalent g-value, line width and peak height, suggesting that the detected radicals in roasted beef were derived from lyophilization rather than roasting; additionally, no stable radicals were generated in beef during roasting.

To detect the unstable radicals formed in roasted beef, spin-trapping of PBN was used in the present work. A typical triplet of doublet signals arising from PBN-radical spin adducts was produced in the liquid analytical sample of roasted beef as shown in Figure 1b. Additionally, because of no detected radical signal in the ESR measurement performed with a blank buffer system of only MES, PBN and ethanol (ESR spectrum not shown), and with a control of raw beef group incubated at 55 °C (Figure 1b), it is reasonable to infer that the PBN trapped radicals in roasted beef were roasting-induced radicals. This radical species was characterized by a *g*-value of 2.0051 and hyperfine coupling constants of a_N_ = 16.1 G and a_H_ = 3.3 G, suggesting that the PBN trapped radicals were alkyl radical species [26], which were the main radicals produced in the lipid oxidation process [19].

### 3.2. Effects of the Heating Method and Time on Radical Formation

The radical formation of heated beef during roasting, grilling and barbecuing at 200 °C was monitored over time, and the results are presented in Table 1. ANOVA indicates significant effects of heating methods, heating time, and the interaction between heating methods and heating times on the radical content (*p* < 0.001). In general, during the heating process, grilled beef had the highest radical content, followed by roasted beef and barbecued beef (Table 1). This result likely implies that the oxidation rate of grilled beef was higher than those of roasted and barbecued beefs. The differences in the radical content among the three heating methods might result from the distance between the beef sample and the heat source. During pan grilling, beef is in contact with the pan surface; thus the rate of heat conduction is faster, and the temperature and radical content of the beef samples quickly increases. In contrast, the beef on the grill was 5 cm away from the lower heating tube in the electric barbecuing, and the beef was 10 cm away from the upper and lower heating tubes in the oven in our work.

It is clear from Table 1 that the radical content significantly increased during heating from 10 to 20 min and decreased at 25 min. During heating from 10 to 20 min, the mean radical contents significantly increased (*p* < 0.05) from 10.54 × 10^15^ to 41.01 × 10^15^ spin/g in roasted beef, from 26.83 × 10^15^ to 46.53 × 10^15^ spin/g in grilled beef, and from 7.64 × 10^15^ to 32.25 × 10^15^ spin/g in barbecued beef, which indicated that the radical generation rate during the initiation and propagation phases was faster than the radical decay rate during the termination phase in the process of lipid oxidation. The radical contents in roasted, grilled, and barbecued beefs all peaked at 20 min. When the heating time was further extended to 25 min, significant decreases of 26.87%, 15.54% and 12.24% (*p* < 0.05) in the radical contents were observed for roasted, grilled and barbecued beef, respectively. This result was consistent with Chen et al. [27], who found that higher radical contents contributed to higher decay rates of radicals through recombination reactions. Reactions among lipid radicals lead to the formation of nonradical products such as aldehydes, alkanes and conjugated dienes [7], which thus contributed to the decrease in radical content at 25 min.

### 3.3. Effects of the Heating Temperature and Water Content on Radical Formation

**Figure 2:**
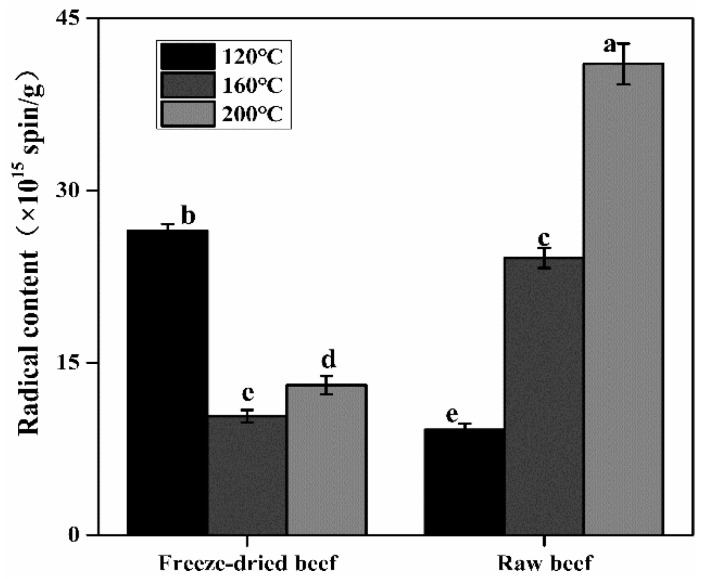
Radical contents of the freeze-dried and raw beefs roasted at different temperatures for 20 min. Means with different letters differ significantly (*p* < 0.05).

The radical formation at different heating temperatures for 20 min was investigated by using freeze-dried and raw beef. ANOVA indicates significant effects of heating temperatures, water contents, and the interaction between heating temperatures and water contents on the radical content (*p* < 0.001, as seen in supplement Table S1). As shown in Figure 2, by increasing the heating temperature, the radical content in the raw beef showed a significant increase (*p* < 0.05) from 9.16 × 10^15^ spin/g to 41.01 × 10^15^ spin/g, which was consistent with the general knowledge that higher temperature contributes to higher radical content. This increasing trend indicated that the radical generation rate of these raw beef samples was faster than the radical recombination reaction rate. However, a significant decrease (*p* < 0.05) in freeze-dried beef from 26.48 × 10^15^ spin/g to 10.35 × 10^15^ spin/g was observed during roasting from 120 to 200 °C, indicating that radical formation in freeze-dried beef had been in the decay stage and that more nonradical oxidation products had been produced. This decreasing trend of radical content could be attributed to the lack of water during roasting; thus the internal temperature of the freeze-dried beef increased quickly, resulting in the accelerated development of lipid peroxidation and a decay phase that began earlier at a higher temperature. Thus, at 160 and 200 °C the radical content in freeze-dried beef was lower than that in raw beef (Figure 2), implying that the lipid nonradical oxidation product content was higher than that in raw beef. Labuza et al. [28] reviewed that dried foods with a moisture content that was too low (less than 2% to 3%) became very susceptible to oxidation, while a high water content slowed lipid oxidation. A similar result was observed here by monitoring radical formation in freeze-dried beef with 1.53% water content and in raw beef with 78.63% water content. Therefore, considering the effect of water content on lipid oxidation as evaluated by radical reaction, it could be suggested that a higher water content should be retained in meat during roasting.

### 3.4. Effects of Antioxidants on Radical Formation among Meat Species

**Figure 3:**
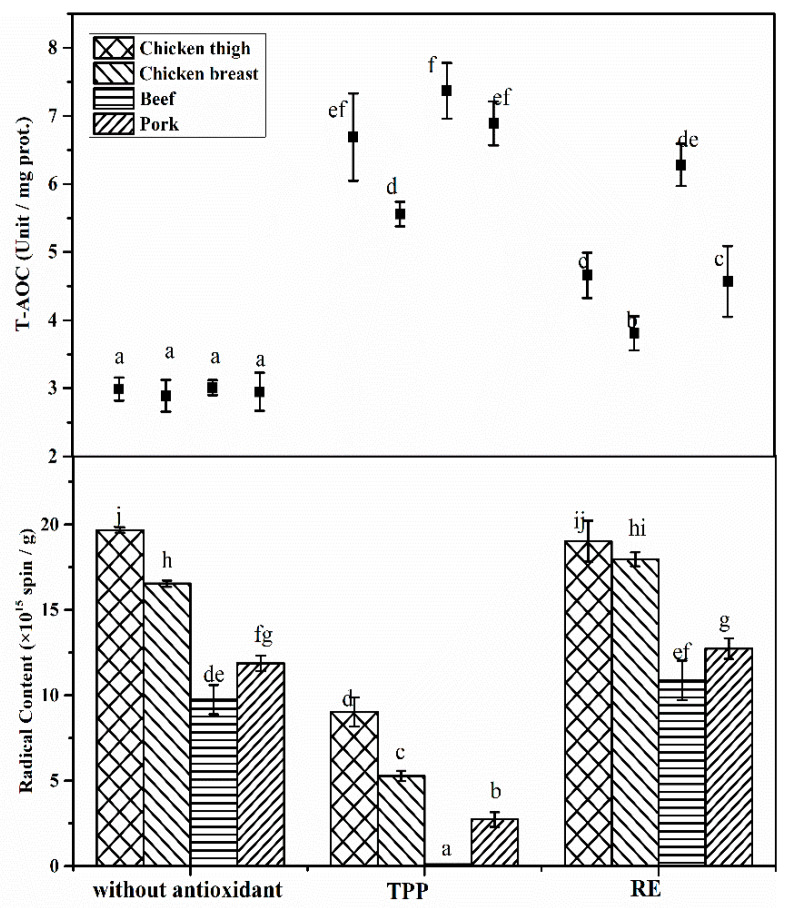
Index of radical formation and total antioxidant capacity (T-AOC) in roasted chicken thigh, chicken breast, pork and beef with or without the addition of antioxidants. Means with different letters are significantly different (*p* < 0.05).

Figure 3 shows the results regarding the level of radical formation among roasted beef, pork, chicken breast, and thigh with and without antioxidants. All roasted meat groups produced similar triplets of doublet structures with the same *g*-value and hyperfine coupling constants in ESR spectra, which facilitated a direct comparison among the effects, suggesting that the same radicals were trapped in the four meat species during the roasting process [17]. Roasted chicken thigh had a higher radical content than roasted chicken. A similar result was reported by Bragagnolo et al. [16] in pressurized chicken breast and thigh. Roasted pork showed higher radical formation than roasted beef, but both of them were significantly lower than roasted chicken thigh and breast (*p* < 0.05). This observation reflects a clear radical formation potential of the roasted meat species and indicates a difference in the susceptibility to lipid oxidation among the meat species after roasting, which is in line with the previous result of oxidation stability as evaluated by TBARS [29]. Radical formation from lipid peroxidation occurs readily in the presence of unsaturated fatty acids and iron catalysts. The higher radical content in roasted chicken thigh could be explained by the higher PUFA content (as seen in supplement Table S2), which could exponentially increase the amount of lipid radicals [30]. Beef and pork have higher myoglobin contents than chicken muscle (as seen in supplement Table S2), but the prooxidant effect of the free ionic iron depends on the presence of reducing compounds in the meat; for example, ascorbic acid reduces ferric iron [31]. However, in our work, roasting could inactivate these heat-labile reducing substances, disrupting the reversible reaction between ferric and ferrous iron and resulting in a limited positive influence of the free ionic iron on lipid peroxidation.

Figure 3 clearly illustrates the effects of TPP and RE on radical formation in the roasted meat species. As expected, with the addition of TPP to meat patties, the T-AOCs of roasted meats were significantly improved, and radical formation was efficiently decreased compared to that without antioxidants (*p* < 0.05). Among all meat species containing TPP, roasted beef was found to have the highest T-AOC as well as the lowest radical formation. In contrast, although the T-AOCs were significantly improved in roasted meats (*p* < 0.05), the addition of RE did not affect significant decreases in radical formation for all meat species (*p* > 0.05). RE has been shown to inhibit radical formation in dehydrated chicken meat [32] and high pressure chicken meat [16]. However, the addition of RE was 0.1% in these previous studies. In the present work, the addition of RE was only 0.03%; thus, the lack of an inhibitory effect of RE on radical formation in roasted meats could be attributed to the inadequate dose that could be added. A similar result was reported by Bolumar et al. [33], who found that the 0.025% addition of RE resulted in no significant decrease in radical formation in high pressure beef. Additionally, Beltran et al. [34] observed that the 0.04% RE addition had no protective effect on lipid oxidation as evaluated by TBARS in cooked chicken breast.

Villalobos-Delgado et al. [35] reviewed the differences between primary antioxidants, which are also called chain-break antioxidants by scavenging lipid radicals to break the oxidation process, and secondary antioxidants, which are also called preventive inhibitors by such as chelating prooxidant metal ions to limit the radical initiation step in the oxidation process. In the present work, 0.03% RE showed little inhibition of lipid radical formation but significantly increased T-AOC, suggesting that the antioxidant activity of RE here could be mainly attributed to limiting the degree of oxidation. In contrast, with respect to the significant decreases in lipid radical content in roasted meat, 0.03% TPP is also suggested to effectively and directly target lipid radicals to protect against the propagation of oxidation. Therefore, TPP could be a good choice as a natural antioxidant used in roasted meat products to scavenge carbon-centered radicals of lipids.

## 4. Conclusions

This work demonstrated for the first time the effect of water content in roasted meat on lipid alkyl radical formation by using ESR. It was clearly shown that a high water content retarded radical generation, suggesting that retaining proper water in meat during roasting is recommended. Moreover, the addition of 0.03% TPP to roasted meat is a practical and effective way to decrease lipid radical formation. Lipid radical detection could be used to estimate the main stage of the lipid oxidation process and evaluate the main antioxidant mechanism of antioxidants.

## Figures and Tables

**Table 1:** The lipid radical content (× 10^15^ spin/g) in roasted, grilled, and barbecued beef over time at 200 °C.

Cooking Methods	Time (min)				P_T_	P_M_	P_T_ × P_M_
	10	15	20	25			
Roast	10.54 ± 1.04 ^b^	34.62 ± 1.56 ^g^	41.01 ± 1.78 ^i^	29.99 ± 1.64 ^e,f^	***	***	***
Grill	26.83 ± 1.57 ^d^	38.08 ± 1.69 ^h^	46.53 ± 1.83 ^j^	39.30 ± 1.72 ^h,i^			
Barbecue	7.64 ± 0.96 ^a^	18.01 ± 1.44 ^c^	32.25 ± 1.52 ^f,g^	28.30 ± 1.61 ^d,e^			

Note: Results are represented as the mean values ± standard deviations (*N* = 3). ^a–j^ Means with different superscript letters differ significantly (*p* < 0.05). P_T_ means *p* value of cooking time effect; P_M_ means *p* value of cooking method effect; P_T_ × P_M_ means *p* value of interaction between cooking time and cooking method effects. *** means *p* < 0.001.

## Supplementary Materials

The following are available online at https://www.mdpi.com/2304-8158/9/5/572/s1, Table S1: Analysis for different temperature, water content, and their interaction on radical content. Table S2: Characterizations of the raw beef, pork, chicken breast and thigh by the total lipid, total myoglobin, fatty acid and radical concentrations profile.

## References

[1] Miyakawa H., Mason R.P., Jiang J., Kadiiska M.B. (2009). Lipid-derived free radical production in superantigen-induced interstitial pneumonia. Free Radic. Biol. Med..

[2] Ku H.H., Brunk U.T., Sohal R.S. (1994). Relationship between mitochondrial superoxide and hydrogen peroxide production and longevity of mammalian species. Free Radic. Biol. Med..

[3] Taub I.A. (1984). Free radical reactions in food. J. Chem. Educ..

[4] Cämmerer B., Kroh L.W. (2009). Shelf life of linseeds and peanuts in relation to roasting. LWT-Food Sci. Technol..

[5] Yeretzian C., Pascual E.C., Goodman B.A. (2012). Effect of roasting conditions and grinding on free radical contents of coffee beans stored in air. Food Chem..

[6] Szöcs F. (2002). Free radicals in wheat seeds studied by electron spin resonance. J. Food Sci..

[7] Falowo A.B., Fayemi P.O., Muchenje V. (2014). Natural antioxidants against lipid–protein oxidative deterioration in meat and meat products: A review. Food Res. Int..

[8] Min S., Patra J.K., Shin H.S. (2018). Factors influencing inhibition of eight polycyclic aromatic hydrocarbons in heated meat model system. Food Chem..

[9] Yu C., Shao Z., Liu B., Zhang Y., Wang S. (2016). Inhibition of 2-amino-1-methyl-6-phenylimidazo 4,5-b pyridine (PhIP) formation by alkoxy radical scavenging of flavonoids and their quantitative structure-activity relationship in a model system. J. Food Sci..

[10] Zhou Y., Wang Q., Wang S. (2020). Effects of rosemary extract, grape seed extract and green tea polyphenol on the formation of N-nitrosamines and quality of western-style smoked sausage. J. Food Process. Preserv..

[11] Andrade M.A., Ribeiro-Santos R., Guerra M., Sanches-Silva A. (2019). Evaluation of the oxidative status of salami packaged with an active whey protein film. Foods.

[12] Embuscado M.E., Shahidi F. (2015). Herbs and spices as antioxidants for food preservation. Handbook of Antioxidants for Food Preservation.

[13] Orak H., Yagar H., Isbilir S., Demirci A., Gumus T. (2013). Antioxidant and antimicrobial activities of white, green and black tea extracts. Acta Aliment..

[14] Martinez L., Castillo J., Ros G., Nieto G. (2019). Antioxidant and antimicrobial activity of rosemary, pomegranate and olive extracts in fish patties. Antioxidant.

[15] Belles M., Alonso V., Roncales P., Beltran J.A. (2017). Effect of borage and green tea aqueous extracts on the quality of lamb leg chops displayed under retail conditions. Meat Sci..

[16] Bragagnolo N., Danielsen B., Skibsted L.H. (2007). Rosemary as antioxidant in pressure processed chicken during subsequent cooking as evaluated by electron spin resonance spectroscopy. Innov. Food Sci. Emerg. Technol..

[17] Davies M.J. (2016). Detection and characterisation of radicals using electron paramagnetic resonance (EPR) spin trapping and related methods. Methods.

[18] Escudero R., Valhondo M., Ordoñez J.A., Hoz L.D.L., Cabeza M.C., Velasco R., Camberoa M.I. (2012). Electron spin resonance (ESR) spectroscopy study of dry-cured ham treated with electron-beam. Food Chem..

[19] Xie Y., Jiang S., Li M., Guo Y., Cheng Y., Qian H., Yao W. (2019). Evaluation on the formation of lipid free radicals in the oxidation process of peanut oil. LWT-Food Sci. Technol..

[20] Zang S., Tian S., Jiang J., Han D., Yu X., Wang K., Li D., Lu D., Yu A., Zhang Z. (2017). Determination of antioxidant capacity of diverse fruits by electron spin resonance (ESR) and UV–vis spectrometries. Food Chem..

[21] National Health and Family Planning Commission of the People’s Republic of China (2016). National Food Safety Standard—Determination of Moisture in Foods.

[22] Carlsen C.U., Andersen M.L., Skibsted L.H. (2001). Oxidative stability of processed pork. assay based on esr-detection of radicals. Eur. Food Res. Technol..

[23] Bolumar T., Skibsted L.H., Vibeke O. (2012). Kinetics of the formation of radicals in meat during high pressure processing. Food Chem..

[24] Abbas K., Babić N., Peyrot F. (2016). Use of spin traps to detect superoxide production in living cells by electron paramagnetic resonance (EPR) spectroscopy. Methods.

[25] Dodd N.J., Swartz H.M. (1984). The nature of the ESR signal in lyophilized tissue and its relevance to malignancy. Br. J. Cancer.

[26] Zhang J.C., Zhao B.L., Guo Y.J., Xin W.J. (1991). Evidence for L·against LOO being spin-trapped by 4-POBN during the reaction of Fe^2+^-induced lipid peroxidation. Appl. Magn. Reson..

[27] Chen H., Wang Y., Cao P., Liu Y. (2017). Effect of temperature on thermal oxidation of palmitic acid studied by combination of EPR spin trapping technique and SPME-GC–MS/MS. Food Chem..

[28] Labuza T.P., Dugan L.R. (1971). Kinetics of lipid oxidation in foods. CRC Crit. Rev. Food Technol..

[29] Min B., Nam K.C., Cordray J., Ahn D.U. (2008). Endogenous factors affecting oxidative stability of beef loin, pork loin, and chicken breast and thigh meats. J. Food Sci..

[30] Wagner B.A., Buettner G.R., Burns C.P. (1993). Free radical-mediated lipid peroxidation in cells: Oxidizability is a function of cell lipid bis-allylic hydrogen content. Biochemistry.

[31] Min B., Ahn D.U. (2005). Mechanism of lipid peroxidation in meat and meat products—A review. Food Sci. Biotechnol..

[32] Nissen L.R., Månsson L., Bertelsen G., Huynh-Ba T., Skibsted L.H. (2000). Protection of dehydrated chicken meat by natural antioxidants as evaluated by electron spin resonance spectrometry. J. Agric. Food Chem..

[33] Bolumar T., Andersen M.L., Orlien V. (2014). Mechanisms of radical formation in beef and chicken meat during high pressure processing evaluated by electron spin resonance detection and the addition of antioxidants. Food Chem..

[34] Beltran E., Pla R., Yuste J., Mor-Mur M. (2004). Use of antioxidants to minimize rancidity in pressurized and cooked chicken slurries. Meat Sci..

[35] Villalobos-Delgado L.H., Mateo J., Caro I., Leal Ramos M.Y., Mendez N.G., Cansino R.G., González-Mondragón E.G., Galanakis C.M. (2019). Natural antioxidants in fresh and processed meat. Sustainable Meat Production and Processing.

