# Supplementary material for: Formation and Inhibition of Lipid Alkyl Radicals in Roasted Meat
Source: Foods. 2020 May 4;9(5):572. doi: 10.3390/foods9050572 (PMC7278865; doi:10.3390/foods9050572)
Supplement: Supplementary file 1 [file foods-09-00572-s001.docx]

**Table 1.** Two-way analysis for different temperature, water content, and their interaction on radical content.

| **Effect Tested** | **Radical content** |
| --- | --- |
| Temperature (T) |  |
| 120℃ | 17.82 ± 9.50 ^a^ |
| 160℃ | 17.24 ± 7.58 ^a^ |
| 200℃ | 27.03 ± 15.37 ^b^ |
| Significance | *** |
| Water content (W) |  |
| Freeze-dried beef | 16.62 ± 7.50 ^A^ |
| Raw beef | 24.77 ± 13.84 ^B^ |
| Significance | *** |
| Interaction (T × W) | *** |

Note: *** = p < 0.001; different small letters (a-b) in the temperatures and capital letters (A-B) in the water contents mean significant differences (p < 0.05).

**Table 2.** Characterizations of the raw beef, pork, chicken breast and thigh by the total lipid, total myoglobin, fatty acid and radical concentrations profile.

| **Characterizations** | **Beef** | **Pork loin** | **Chicken breast** | **Chicken thigh** | **SEM** |
| --- | --- | --- | --- | --- | --- |
| Total myoglobin (mg / kg) | 7.69 ^c^ | 6.40 ^b, c^ | 3.77 ^a^ | 5.68 ^b^ | 0.38 |
| Total Lipid (%) | 1.77 ^a^ | 3.26 ^c^ | 1.56 ^a^ | 5.03 ^d^ | 0.22 |
| Percent of total lipid | | | | | |
| SFA | 43.72 ^c^ | 37.93 ^b^ | 33.02 ^a^ | 31.77 ^a^ | 0.70 |
| MUFA | 45.20 ^c^ | 35.88 ^b^ | 33.47 ^a, b^ | 31.01 ^a^ | 0.68 |
| PUFA | 11.09 ^a^ | 26.30 ^b^ | 33.52 ^c^ | 37.23 ^c^ | 1.32 |

^a-d^ Means with different letters within the same row are significant different (*p* < 0.05). SFA = saturated fatty acid; MUFA = monounsaturated fatty acid; PUFA = polyunsaturated fatty acid; SEM = standard error of the means.
